# 胸腔镜肺癌肺叶切除术后16 F较28 F胸腔引流管应用的临床优势

**DOI:** 10.3779/j.issn.1009-3419.2015.08.08

**Published:** 2015-08-20

**Authors:** 梅 杨, 骏 樊, 红霞 周, 恒 杜, 舫 邱, 琳 林, 伦旭 刘, 国卫 车

**Affiliations:** 610041 成都，四川大学华西医院胸外科 Department of Thoracic Surgery, West China Hospital, Sichuan University, Chengdu 610041, China

**Keywords:** 胸腔引流管大小, 胸腔镜肺叶切除术, 肺肿瘤, Chest tube size, VATS lobectomy, Lung neoplasms

## Abstract

**背景与目的:**

微创胸外科术后管理仍延用开放术后的方式，尤其是胸腔引流管的术后管理，本研究探讨胸腔镜（video-assisted thoracic surgery, VATS）肺叶切除术后应用胸腔引流管（16 F）对切口愈合延迟的影响，是否因引流管管径小而导致相关并发症的增多。

**方法:**

选取2014年2月-2014年5月四川大学华西医院连续收治的163例肺癌行VATS肺叶切除术，分别应用引流管28 F（75例）和16 F（88例），分析术后胸腔积气、积液、皮下气肿、引流管持续时间、术后住院日、术后引流管拆线时间和切口愈合率。

**结果:**

平均引流量和心律失常发生率在16 F组[(365±106) mL, 14.67%]明显低于28 F组[(665±217) mL, 4.5%]（*P*=0.030, 1, *P*=0.047）；术后胸腔积气、积液和皮下气肿在28 F组发生率（4.00%, 0.0%, 7.50%）与16 F组（4.50%, 3.41%, 6.82%）均无统计学差异（*P* < 0.999, *P*=0.025, 3, *P*=0.789）；引流管持续时间及术后平均住院日在16F组[(22.1±11.8) h, (4.23±0.05) d]与28 F组[(28.4±16.12) h, (4.57±0.16) d]均无统计学差异（*P*=0.12, *P*=0.078）；引流管拆线时间在16 F组（7.05±2.11）d明显短于28 F组（14.33±3.87）d（*P*=0.034）；切口一级愈合率在16 F组（95.45%）明显高于28 F组（77.73%）（*P*=0.039）。

**结论:**

胸腔镜肺叶切除术后16 F和28 F引流临床效果相当，而16 F有助于引流管口快速愈合。

## Abstract (en)

**Background and objective:**

Post-operation management of minimally invasive thoracic surgery is similar to that of open surgery, especially on the drainage tube of the chest. The aim of this study is to compare the advantages of using 16 F versus 28 F chest tubes in video-assisted thoracoscopic surgery (VATS) lobectomy of lung cancer.

**Methods:**

Data from 163 patients (February-May 2014) who underwent VATS lobectomy of lung cancer with insertion of one chest drain (16 F or 28 F) were analyzed. The following post-operative data were evaluated: primary healing of tube incision, CXR abnormalities (pneumothorax, fluid, atelectasis, subcutaneous emphysema, and hematoma), drainage time, new drain insertion, and wound healing at the site of insertion.

**Results:**

A total of 75 patients received 28 F chest tubes, and 88 patients received 16 F chest tubes. Both groups were similar in age, gender, comorbidities, and pathological evaluation of resection specimens. After adjustment, no statistically significant difference was found between the two groups in relation to tube-related complications including residual pneumothoraces (4.00% *vs* 4.44%; *P* > 0.999), subcutaneous emphysema (8.00% *vs* 6.67%; *P*=0.789), retained hemothorax (0 *vs* 41%, *P*=0.253), and drainage time [(28.4±16.12) h *vs* (22.1±11.8) h; *P*=0.120)] The average total drainage volume and rrhythmia rates of the 16 F group [(365±106) mL, 14.67%)] was less than that of the 28 F group [(665±217) mL, 4.5%; *P*=0.030, *P*=0.047]. The rates of primary healing at the site of insertion in the 16 F group (95.45%) was higher than that in the 28 F group (77.73%, *P*=0.039). A significant difference was found on the post-operative length of stay of the two groups [(4.23±0.05) d *vs* (4.57±0.16) d, *P*=0.078].

**Conclusion:**

The use of 16 F chest tube for VATS lobectomy of patients with lung cancer did not affect the clinically relevant outcomes tested. However, 16 F chest tube facilitated faster wound healing at the site of insertion.

肺叶切除术后行胸腔闭式引流已是常规和大家共识，但现实的问题是肺癌手术方式和术中（麻醉和处理）相关措施已取得了巨大进步，而术后管理（如引流管和尿管等）却没有变化。基于20世纪肺叶切除术的手术方式（主要是开放手术）和术中处理（超声刀和切割缝合器应用少），术后应用胸腔引流管的目的主要是预防胸腔积气或积液和促进肺复张，以减少相关并发症^[1]^。但是近10年来，腔镜肺叶切除术已成为肺叶切除术（超声刀和切割缝合器大量应用）的主要术式，但是术后仍延用36 F、32 F或28 F引流管进行引流，目前大家倾向于28 F，但是28 F PVC引流管有助于术后肺复张，但仍需要术后留置预置线以固定引流管，而预置线却给患者带来极大的不适，如疼痛、活动障碍和引流管口延迟愈合等^[2, 3]^，而16号引流管却不需要预置线，只需用切口缝线固定，则明显促进伤口愈合。胸腔镜肺叶切除术后引流管的应用双管和单管均显示出相同的引流效果^[4, 5]^，而引流管管径大小对术后引流临床效果的研究，尚没有相关报道，我们前瞻性地分析了16 F和28 F引流管在肺叶切除术后的引流效果及相关临床资料，初步探讨引流管径大小对患者术后恢复的影响。

## 1 资料与方法

### 1.1 临床资料

连续分析2014年2月-2014年5月在四川大学华西医院胸外科行胸腔镜肺癌肺叶切除术的患者216例。纳入标准：①病理学检查诊断为原发性肺癌；②手术方式为电视辅助胸腔镜手术（video-assisted thoracic surgery, VATS）肺叶（单叶或双叶）+系统淋巴结清扫术；③临床资料完整。排除标准：①病历资料不完整；②临床资料不完整；③开放手术的肺癌患者或全肺切除患者；④术后出血或持续漏气需要再次手术的患者。最终纳入患者163例，28 F组75例，16 F组88例。术后分期采用国际抗癌联盟（Union for International Cancer Control, UICC）（2009）肺癌分期标准。患者临床特征（表 1）及实验室检查指标（表 2）。

**1:** 两组患者临床特征 The characteristics of the patients

Characteristics	28 F group (*n*=75)	16 F group (*n*=88)	*P*
Gender			
Male	35	35	
Female	40	53	
Age (yr)	53.18±14.73	56.62±12.62	0.052
Male	52.79±14.53	57.17±12.73	0.247
Female	53.43±15.08	55.91±11.77	0.09
Smoking (%)	35.62% (26/75)	46.59% (41/88)	0.200
Concomitant diseases	44%（33/75）	45.45% (40/88)	1.005
Tuberculosis	6.67% (5/75)	0.00% (0/88)	0.245
Asthma	1.33% (1/75)	2.27% (2/88)	0.451
Nasitis	0.00% (0/75)	2.27% (2/88)	0.502
Injury	2.67% (2/75)	3.41% (3/88)	< 0.999
Lung bubble	2.67% (2/75)	20.45% (18/88)	< 0.999
Hypertension	20.00% (15/75)	12.50% (11/88)	0.845
Diabetes	6.67% (5/75)	2.27% (2/88)	0.296
Coronary disease	4.00% (3/75)	2.27% (2/88)	0.658
Histology			
Squamousl carcinoma	25.33% (19/75)	20.45% (18/88)	0.983
Adenocarcinoma	66.67% (50/75)	70.45% (62/88)	0.201
Small cell carcinoma	4.00% (3/75)	2.27% (2/88)	0.653
Others	4.00% (3/75)	6.81% (6/88)	0.132
TNM stage			
Ⅰ	28.00% (21/75)	37.50% (33/88)	0.133
Ⅱ	65.33% (49/75)	51.14% (45/88)	0.082
Ⅲ	6.67% (5/75)	9.09% (8/88)	0.210
Ⅳ	0.00% (0/75)	2.27% (2/88)	1.002

**2:** 两组患者实验室检查相关指标 Comparison of laboratory index of correlation between 16 F group and 28 F group

Item	16 F group	28 F group	*P*
RBC (×10^12^/L)	4.58±0.50	4.68±1.27	0.260
Hb (g/L)	138.18±19.70	137.03±16.27	0.871
WBC (×10^9^/L)	5.89±1.94	5.73±1.56	0.218
PLT (×10^9^/L)	172.68±61.48	176.28±68.00	0.542
Albumin (g/L)	43.56±5.96	42.96±6.40	0.617
Globin (g/L)	27.05±4.92	27.45±5.11	0.302
Blood glucose (mmol/L)	5.31±0.91	5.37±0.80	0.380
FEV1%	104.81±16.61	92.78±23.14	0.059
DLco	21.81±6.23	20.48±7.03	0.946
FVC%	113.28±16.49	101.94±20.07	0.425
MVV%	107.89±21.59	100.98±26.96	0.415
RBC: red blood cell; Hb：hemoglobin; WBC：white blood cell; PLT: blood platelet; FEV1：forced expiratory volume in one second; DLco: diffusing capacity for carbon monoxide; MVV: maximum voluntary ventilation; FVC: forced vital capacity.			

### 1.2 方法

手术方法：VATS手术方式应用单向式胸腔镜肺叶切除法+系统淋巴结清扫^[6]^。系统淋巴结清扫左侧必须清扫第5、6、7、8、9、10组淋巴结，右侧包括第2、3、4、7、8、9、10组淋巴结。引流管应用方法：胸腔引流管统一选用扬州市邗江华飞医疗器件厂生产的一次性使用硅橡胶28 F和16 F引流管，均应用单引流管是将16 F或28 F硅橡胶引流管从7肋间镜孔经后胸胸壁向上直达胸顶，不需另加侧孔，两组患者术后均应用相同的水封引流瓶，且均不加用负压吸引^[5]^；16 F组不加用留置线，28 F需应用留置线。

### 1.3 术后处理

拔管后均鼓励患者咳嗽，必要时刺激患者咳嗽。术后第2天均行胸部照片，若无漏气且每天引流量小于300 mL，肺已复张则拔除引流管。术后疼痛处理均应用镇痛泵（5 mg loading dose followed by 1.0 mg/h-1.5 mg/h），均早期促使患者下床活动。必要时应用非甾体类止痛药（泰勒宁或芬必得）。镇痛泵于引流管拔除的同时也一起停止^[5]^。

### 1.4 观察指标

临床特征：年龄、性别、病理和分期。术后并发症^[7]^：包括腹泻、过敏反应、皮下气肿、心律失常、小便失禁、术后胸腔积气（胸部X线片提示：胸腔积气 > 30%）、术后胸腔积液（胸部X线片提示：胸腔积液中量以上）、肺部感染^[8]^（①明确的病原学证据；②影像学提示肺不张或大片状影；③发热；④白细胞总数大于10, 000/mL或15, 000/mL）。手术后观察胸腔引流量、引流时间、术后住院时间、再次置管等、术后引流管拆线时间和引流管口一级愈合率等。

### 1.5 统计学方法

统计分析采用SPSS 16.0软件包，计数资料采用实际例数及百分比表示，计量资料采用均数±标准差（Mean±SD）表示。计数资料的比较采用χ^2^或*Monte-Carlo*确切概率法进行分析，计量资料比较采用两独立样本的*t*检验。*P* < 0.05为差异有统计学意义。

## 2 结果

### 2.1 两组患者术后并发症分析

两组患者术后心律失常发生率在28 F组（14.67%）明显高于16 F组（4.50%）（*P*=0.047）；术后并发症如术后胸腔积气与积液、皮下气肿、再次置管率和肺部感染两组之间均无统计学差异；而过敏反应、腹泻、乳糜胸、肺栓塞和小便失禁在两组间均无统计学差异（表 3）。

**3:** 两组术后并发症分析 Comparison of complication between 16 F group and 28 F group

	28 F group	16 F group	*P*
Arhythmia	14.67% (11/75)	4.50% (4/88）	0.047
Aerothorax	4.00% (3/75)	4.50% (4/88）	1.000
Hemothorax	0.00% (0/75)	3.41% (3/88）	0.253
Subcutaneous emphysema	7.50% (6/75)	6.82% (6/88）	0.789
Lung infection	0.00% (0/75)	0.00% (0/88)	1.230
Reinserted chest tube (%)	1.33% (1/75)	3.41% (3/88）	0.531
Diarrhea	5.33% (4/75)	2.28% (2/88)	0.076
Aconuresis	2.67% (2/75)	5.68% (5/88)	0.104
Anaphylaxis	2.67% (2/75)	2.28% (2/88)	0.631
Chylous hydrothorax	2.67% (2/75)	1.14% (1/88)	0.172
Pulmonary embolism	1.33% (1/75)	1.14% (1/88)	0.887

### 2.2 两组患者术后引流临床效果

引流管持续时间及术后平均住院日在16 F组[(22.1±11.8)h, (4.23±0.05)d]与28 F组[(28.4±16.12)h, (4.57±0.16)d[均无统计学差异（*P*=0.12, *P*=0.078）；而术后引流量在28 F组（665.33±217.67）mL明显高于16 F组（365.70±106.23）mL（*P*=0.030）；引流管拆线时间在16 F组（7.05±2.11）d明显短于28 F组（14.33±3.87）d（*P*=0.034）；术后切口一级愈合率在16 F组（88%）明显高于28 F组（17%）（*P*=0.013）（表 4）。

**4:** 两组患者临床引流效果分析 Comparison of clinical effect of drainage between 16 F group and 28 F group

Complication	28 F group	16 F group	*P*
Chest tube duration (h)	28.4±16.12	22.1±11.8	0.120
Postoperative length of stay (d)	4.57±0.16	4.23±0.05	0.078
Drainage volume (mL)	665.33±217.67	365.70±106.23	0.030
Take out stitches of the site of insertion (d)	14.33±3.87	7.05±2.11	0.034
Primary healing at the site of insertion (%)	77.73% (58/75)	95.45% (84/88)	0.039

## 3 讨论

胸腔镜肺叶切除术和淋巴结清扫术已成为肺癌外科治疗的主要方法，术后传统的胸腔引流方法是两根引流管（一根在前上引流气体，另一根在后下引流液体）。这种引流方法（双引流管）过分强调了充分排除胸腔气体和液体、并促使肺复张^[1]^，而忽视了肺叶外科技术（微创肺叶切除术）的进步而应改进的术后管理（各种管道等）和引流管自身的缺点：既不利于患者排痰和肺复张，也不利于患者活动和物理康复训练等^[5, 8, 9]^，这明显降低了胸腔镜肺叶切除术微创效果和快速康复。

近10年来将单管引流应用于VATS或开放肺叶切除术，均取得了与双管引流同样的临床效果，且单引流管在术后舒适度和快速康复中显示出明显优势^[5, 10, 11]^。单管引流在目前的临床应用中大家常用的引流管管径大小为36 F、32 F、28 F、24 F和16 F，而以32 F、28 F为主。32 F和28 F引流管在临床应用中的问题包括：①引流管口周围易渗液或漏气，管径粗不易密闭；②术后均需要留置预置线，以利拔管后封闭引流管口；③预置线结扎后导致皮肤坏死、甚至感染，延迟切口愈合；④引流管口常常以瘢痕方式愈合，需要长期换药而给患者带来极度不适。目前解决上述问题最好的方法是应用管径小的（16 F）引流管且不留预置线，没有预置线可以使引流管口和其他切口同期愈合，同时拆线和达到一级愈合。问题是16 F引流管是否可以达到28 F同样的引流效果且不增加相关并发症呢？我们的研究对连续163例肺癌胸腔镜肺叶手术患者分别应用16 F和28 F引流管的临床应用效果回答了上述问题，也发现了16 F引流管临床应用优势。

16 F和28 F引流管组患者的临床特征相似，伴随疾病也相似，术中及术后处理也无统计学差异。研究表明术后相关并发症术后胸腔积气与积液、皮下气肿、再次置管率在两组间均无统计学差异，且在两组患者中均没有发生肺部感染。其他非相关性并发症如过敏反应、腹泻、乳糜胸、肺栓塞和小便失禁也没有差异。提示16 F和28 F引流管临床引流效果等同，且16 F在临床应用中也不需要因引流管不通畅而反复调整位置。

研究发现16 F组在引流管持续时间及术后平均住院日[(22.1±11.8) h, (4.23±0.05) d）]均没有体现出较28 F组[(28.4±16.12) h, (4.57±0.16) d）]的优势（*P*=0.12, *P*=0.078），但均有减少的趋势。分析其原因是：①两种引流管的引流效果是相同的，且没有增加相关并发症；②我们已有应用单管引流的处理经验，如早期活动、体位引流的应用等^[5]^。若通过术后加强管理，理论上应是可以缩短的，需要进一步研究。

16 F较28 F引流管临床优势主要体现在：①术后心律失常发生率明显降低，以往研究发现，引流管胸腔内部分因位置不当而对心脏有影响，而细引流管从自身硬度和处于胸顶而不易碰触心包；②术后总引流量显著减少，其原因是对胸膜刺激性小而分泌少，还可能和引流时间短有关^[12-14]^；③引流管口拆线时间显著缩短，原因是没有预置线，而使其同其他切口同期愈合；④引流管口一级愈合率显著提高，其原因是预置线常常导致切口皮肤坏死，而使其形成瘢痕，甚至切口感染而延迟恢复；⑤在切口疼痛和患者住院舒适度上也应有优势，我们在随后的研究中报道。我们的研究只是临床连续收治的患者，没有进行严格的随机分组是不足之处，但两组的基线还是一致的。

总之，从研究结果看，16 F引流管可以达到28 F同样的临床效果，且在术后拆线时间和引流管口一级愈合率具有明显优势（图 1），因此临床可以应用细引流管以促进患者术后快速康复。

**1:**
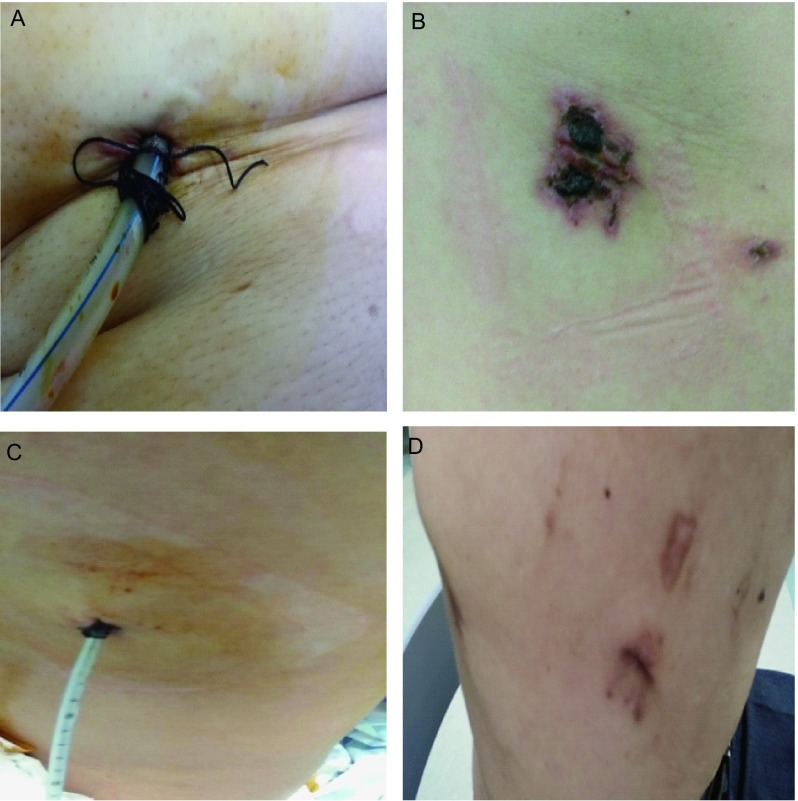
引流管口术后愈合情况。A：28 F引流管术中固定及预置线；B：28 F引流管口1个月后；C：16 F引流管术中固定无预置线；D：16 F引流管口12天后。 Healing at the site of insertion. A: 28 F was fixed in operation and Preset stitch; B: the site of insertion of 28 F after 30 d; C: 16 F was fixed in operation and no Preset stitch; D: the site of insertion of 16 F after 12 d.
